# Low Levels of Vitamin D in Neuromyelitis Optica Spectrum Disorder: Association with Disease Disability

**DOI:** 10.1371/journal.pone.0107274

**Published:** 2014-09-11

**Authors:** Ju-Hong Min, Patrick Waters, Angela Vincent, Hye-Jin Cho, Byung-Euk Joo, Sook-Young Woo, Soo-Youn Lee, Hee-Young Shin, Kwang Ho Lee, Byoung Joon Kim

**Affiliations:** 1 Department of Neurology, Samsung Medical Center, Sungkyunkwan University School of Medicine, Seoul, Korea; 2 Nuffield Department of Clinical Neurosciences, Neuroimmunology group, John Radcliffe Hospital, Oxford, United Kingdom; 3 Biostatistics Team, Samsung Biomedical Research Institute, Seoul, Korea; 4 Department of Laboratory Medicine, Samsung Medical Center, Sungkyunkwan University School of Medicine, Seoul, Korea; 5 Center for Health Promotion, Samsung Medical Center, School of Medicine, Sungkyunkwan University, Seoul, Korea; Medical University Vienna, Center for Brain Research, Austria

## Abstract

Patients with autoimmune disorders often have low levels of 25-hydroxyvitamin D [25(OH)D_3_], which correlates with disability or disease activity. Vitamin D may play a role in neuromyelitis optica (NMO) or NMO spectrum disorder (NMOSD), as an important factor involved in immunological pathways. We investigated the relationship between vitamin D levels and disease related disability and clinical activity in patients with NMOSD. Blood samples from 51 patients with NMOSD who were positive for anti-aquaporin4-antibody (AQP4-ab) and 204 healthy controls were collected for 25(OH)D_3_ measurement. Clinical parameters, including expanded disability status scale (EDSS) score, annualized relapse rate (ARR) and time of blood sampling relative to attack, were determined in patients with NMOSD. We found that 25(OH)D_3_ levels were significantly lower in patients with NMOSD compared to healthy controls. There was no difference between 25(OH)D3 levels in blood samples taken at relapse or remission, and no association between 25(OH)D3 levels and ARR, but there was an inverse correlation between 25(OH)D3 levels and EDSS scores in patients with NMOSD. It remains to be determined whether low vitamin D levels predispose to NMO and/or modify disease severity, or are secondary to neurological disability. In either case the results could also be of relevance to other neurological diseases such as multiple sclerosis as well as NMO.

## Introduction

Vitamin D_3_ is a prohormone produced by the action of ultraviolet (UV) on 7-dehydrocholesterol in the skin. It is metabolized to 25-hydroxyvitamin D [25(OH)D_3_] in the liver, and then converted to the biologically active form, 1,25-dihydroxyvitamin D [1,25(OH)_2_D_3_] in the kidney. The major role of vitamin D in humans is to regulate bone homeostasis and calcium metabolism, but it is also crucial for activating immune defense systems and suppressing immune disease pathology. Vitamin D suppresses B cell proliferation and differentiation to decrease immunoglobulin secretion and affects T cell proliferation and maturation to decrease the numbers of T cells with Th1 and Th17 phenotypes [1], [2]. It also induces T regulatory (Treg) cells to decrease the production of inflammatory cytokines, such as interleukin (IL)-17 and IL-21[3]. These effects of vitamin D were also evidenced in in vivo studies, where vitamin D suppressed disease in animal models of autoimmune diseases, such as multiple sclerosis (MS), systemic lupus erythematosus (SLE), and type 1 diabetes [4]–[6]. Several studies have shown that vitamin D levels are low in patients with autoimmune disorders, including MS, SLE, rheumatoid arthritis (RA), and type 1 diabetes [7]. In addition, vitamin D levels have been reported to be associated with disease disability or activity in these disorders [8]–[10].

Neuromyelitis optica (NMO) is a central nervous system (CNS) autoimmune disorder that preferentially affects the optic nerve and spinal cord, and the discovery of the disease specific antibody, NMO-IgG (anti-aquaporin-4-antibody, AQP4-ab) has widened the spectrum of NMO-related disorders (NMO spectrum disorder, NMOSD) [11], [12]. So far, there are no studies for vitamin D in NMO; however, a recent study describes patients with recurrent transverse myelitis (TM) had lower vitamin D levels compared to those with monophasic TM [13]. They reported also that AQP4-Ab positivity was higher in recurrent TM (61%) than monophasic TM (0%), which may indicate hypovitaminosis D in NMOSD. Here, we analyzed vitamin D levels in patients with NMOSD and healthy controls, and the association of vitamin D levels with clinical parameters in AQP4-Ab positive patients.

## Methods

### Study population

We reviewed data from the CNS demyelinating disease registry of Samsung Medical Center, South Korea that were collected between March 2006 and December 2012. We identified 74 patients with AQP4-ab who had been followed-up for more than 1 year in our hospital. AQP4-ab was measured with a cell-based indirect immunofluorescence assay as previously described [14], [15]. We excluded 3 patients taking vitamin D supplementation at blood sampling, 4 patients who did not meet the revised criteria for NMO or the suggestion of NMOSD [16], [17], and 16 patients who did not have available blood samples for vitamin D measurement. A total of 51 patients with AQP4-ab were ultimately included. The control group included 204 age-, and sex- matched healthy subjects. They had no obvious disorders and were not taking any medications that could affect serum vitamin D levels.

### Ethics Statement

All subjects gave written informed consent prior to participation in the study. This study was approved by Institutional Review Board of Samsung Medical Center **(IRB number: 2011-11-054).**

### Clinical characteristics

All subjects were of Korean ethnicity and had lived in South Korea since birth (latitude: 35′05-37′79). We extracted demographic and clinical characteristics including sex, age at sampling, body mass index (BMI), disease duration (the interval between disease onset and blood sampling), and prednisolone or azathioprine use from the registry database. Season at blood sampling, defined as spring (March to May), summer (June to August), fall (September to November), and winter (December to February) was also acquired and matched to healthy controls. In addition, the patients' expanded disability status scale (EDSS) scores, annualized relapse rates (ARR) and the locations of lesion (optic nerve, spinal cord, and brain) were recorded at blood sampling. Attack was defined as a new worsening of neurological function such as visual loss, limb weakness or sensory symptoms and bladder or bowel dysfunction, lasting for more than 24 hours and not attributable to an identifiable cause. All patients experiencing attacks (within 7 days) had not been treated with intravenous high-dose methylprednisolone before blood sampling. Patients in remission were stable and had not had an attack in the previous 3 months. All except one had taken or had been taking low dose oral prednisolone and/or oral azathioprine as maintenance therapy.

### Vitamin D measurements

25(OH)D_3_ levels were measured with liquid chromatography-tandem mass spectrometry (LC-MS/MS) in our hospital. 25(OH)D_3_ levels <50 nmol/L, 50 to <75 nmol/L) and ≥75 nmol/L were defined as deficient, insufficient, and sufficient, respectively [18].

### Statistical analysis

We analyzed vitamin D3 levels and BMI using Friedman's tests to compare between patients and age-, sex-, and season-matched healthy controls. Regarding the patients, the associations between 25(OH)D_3_ levels and age, sex, season, oral prednisolone use, azathioprine use, and disease duration were analyzed by linear regression analyses. Spearman's correlation analysis was used for the EDSS score (disease disability), and ARR. As there were patients with simultaneous lesions in two locations, the association between lesion location and vitamin D levels was analyzed by generalized estimating equation. Logistic regression analysis was used to assess clinical activity (during attack vs. in remission), as a measure of association.

All potential confounders were included simultaneously in the models so that the analysis could be adjusted. Vitamin D levels and disease durations showed skewed distributions and were therefore transformed using natural log. P-values were corrected by Bonferroni's method due to multiple testing. We did not stratify subjects by latitude because the study included a small sample size, and all the participants lived in the same general area. All statistical analyses were performed using SAS 9.3 (SAS Institute, Cary, NC), and *p* values<0.05 were considered statistically significant.

## Results

### Demographic and clinical features of patients with NMOSD and healthy controls

A total of 51 AQP4-ab-positive patients (mean age, 43.8±12.2 years; female:male = 44∶7) were included in this study and met the diagnostic criteria for NMO (*n* = 26)[16] or the suggestion of NMOSD (*n* = 25) (Table 1).[17] The mean interval from onset to sampling (disease duration) in patients was 5.3±6.1 years (range, 0.01–32.44), EDSS score was 3.8±2.3 (range, 1–9.5) and ARR was 0.8±0.8 (0.0–3.4). The lesions were in the optic nerve (N = 9, 17.6%), spinal cord (N = 3, 64.7%) or brain (N = 12, 23.5%). Samples from 20 patients were taken within 7 days of an attack (relapse samples), and in 31 patients samples were taken more than 3 months from an attack (remission samples).

**Table 1:** Demographic features of patients with neuromyelitis optica spectrum disorder and healthy controls.

	NMOSD (N = 51)	Healthy controls (N = 204)	*p*-value
Definite NMO a (%)	25 (49)		
Age, y b	43.8±12.2	43.6±12.3	0.134
Sex (F:M)	44∶7	176∶28	0.83
Body mass index b	22.8±3.8	22.6±3.0	0.496
Season at sampling			
Spring: Summer: Fall: Winter	16∶13∶8∶14	64∶52∶32∶56	1
Interval from onset to sampling, y b	5.3±6.1		
EDSS score b	3.8±2.3		
ARR b	0.8±0.8		
During relapse: In remission (%)	20 (39.2) ∶31 (60.8)		
Location of lesion (%)			
Optic nerve	9 (17.6)		
Spinal cord	33 (64.7)		
Brain	12 (23.5)		

Abbreviations: NMOSD, neuromyelitis optica spectrum disorder; N, number; y, years; F, female; M, male; EDSS, expanded disability status scale; ARR, annualized relapse rate.

a Patients meeting the revised diagnostic criteria for NMO [16].

b Expressed as mean ± standard deviation.

### Comparison of vitamin D levels between patients with NMOSD and healthy controls

**Figure 1:**
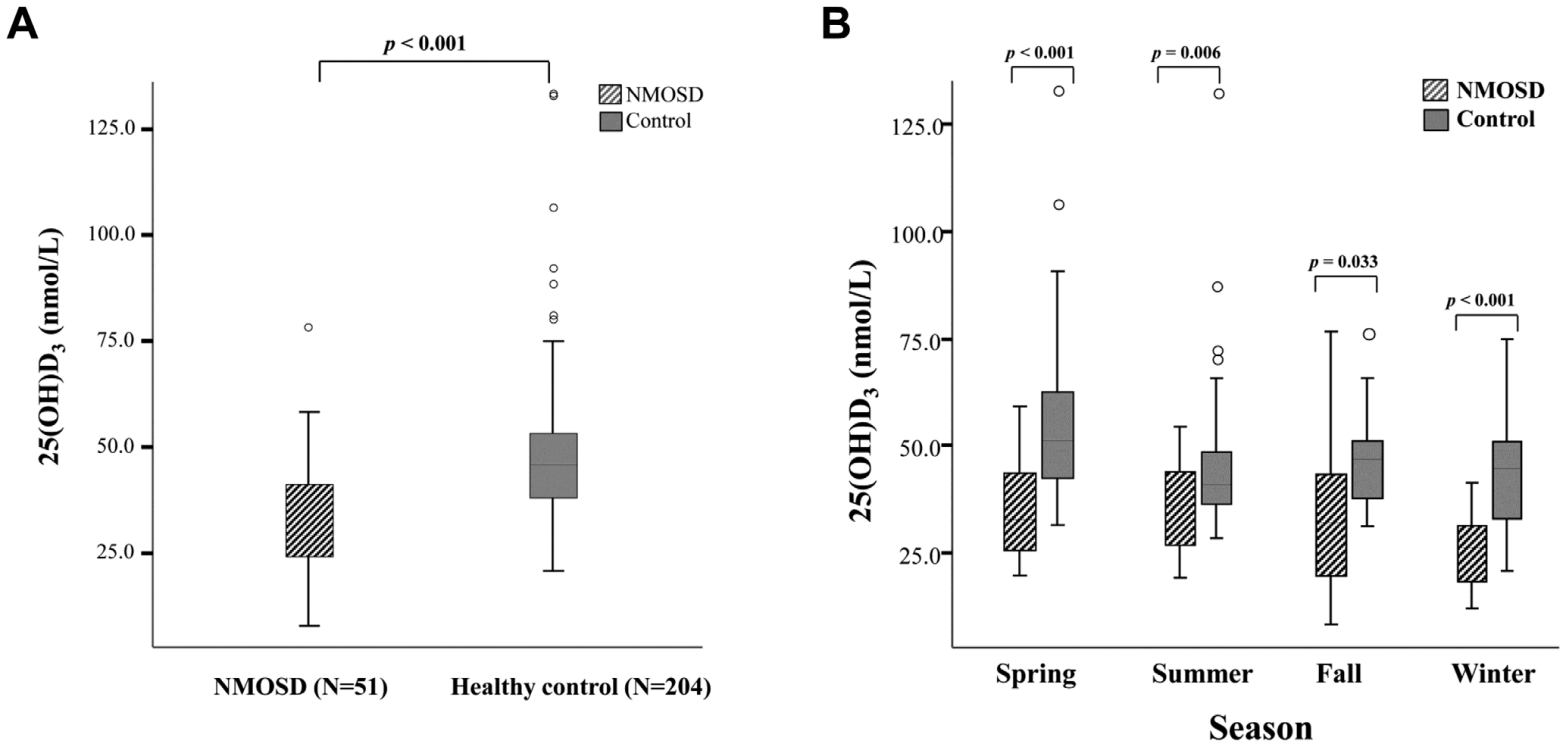
Vitamin D levels in patients with neuromyelitis optica spectrum disorder (NMOSD) (*n* = 51) and healthy controls (*n* = 204). A. Patients with NMOSD showed significantly lower 25(OH)D_3_ levels, compared to healthy controls. B. Vitamin D levels sampled in the spring, summer, fall and winter were all significantly lower in patients with NMOSD than those in healthy controls.

We found that 25(OH)D_3_ levels were significantly lower in patients with NMOSD compared to age-, sex- and season-matched healthy controls (*p*<0.001) (Figure 1A). Among the 51 patients with NMOSD, 47 (92.1%) showed vitamin D deficiency (<50 nmol/L), 3 patients (5.9%) had vitamin D insufficiency (50 to <75 nmol/L), and only 1 patient (2.0%) had a sufficient vitamin D level (≥75 nmol/L). By contrast, 135 (66.2%) of 204 normal controls were considered vitamin D deficient, and 9 (4.4%) had insufficient levels, with 60 (29.4%) having sufficient levels. In the analysis according to the season, vitamin D levels sampled in the spring, summer, fall, and winter were all significantly lower in patients with NMOSD than those in healthy controls (*p*<0.001, *p* = 0.006, *p* = 0.033, and *p*<0.001 respectively) (Figure 1B).

### Variations in the vitamin levels of patients with NMOSD

In patients with NMOSD, 25(OH)D_3_ levels did not differ with regard to sex, age, BMI, or the season at blood sampling. Similarly, oral prednisolone use, azathioprine use, and disease duration did not affect 25(OH)D_3_ levels. Multivariable linear regression analysis revealed that only disease duration was associated with 25(OH)D_3_ levels (*β* = 0.083936, *p* = 0.035; Table 2).

**Table 2:** Multivariable logistic regression analysis for the relationship between 25-hydroxyvitamin D_3_ (25(OH)D_3_) levels and age, sex, body mass index, season, oral prednisolone use, azathioprine use and disease duration in patients with neuromyelitis optica spectrum disorder.

Variable	Coefficienct (β)	Standard Error	t Value	*P* - value
Age (years)	0.003339294	0.0056881	0.59	0.5604
Female^a^	−0.325243895	0.18330196	−1.77	0.0834
Body mass index	0.000411838	0.01760472	0.02	0.9814
Season^b^				0.2854
Spring	−0.235737471	0.16367885	−1.44	0.4722*
Summer	−0.079032037	0.16402617	−0.48	1*
Fall	−0.315022848	0.20050654	−1.57	0.3714*
Oral prednisolone use^c^	−0.056395219	0.2011614	−0.28	0.7806
Azathioprine use^d^	−0.198869907	0.13343339	−1.49	0.1438
Disease duration	0.083935516	0.03850049	2.18	0.035†

The reference categories are male^a^, winter^b^, no use of oral prednisolone^c^, and no use of azathioprine^d^ respectively.

*P-values were corrected by Bonferroni's method due to multiple testing.

† Adjusted for age, sex and BMI, season, oral prednisolone use, and azathioprine use.

### Association between vitamin D and disease disability and clinical disease activity

**Figure 2:**
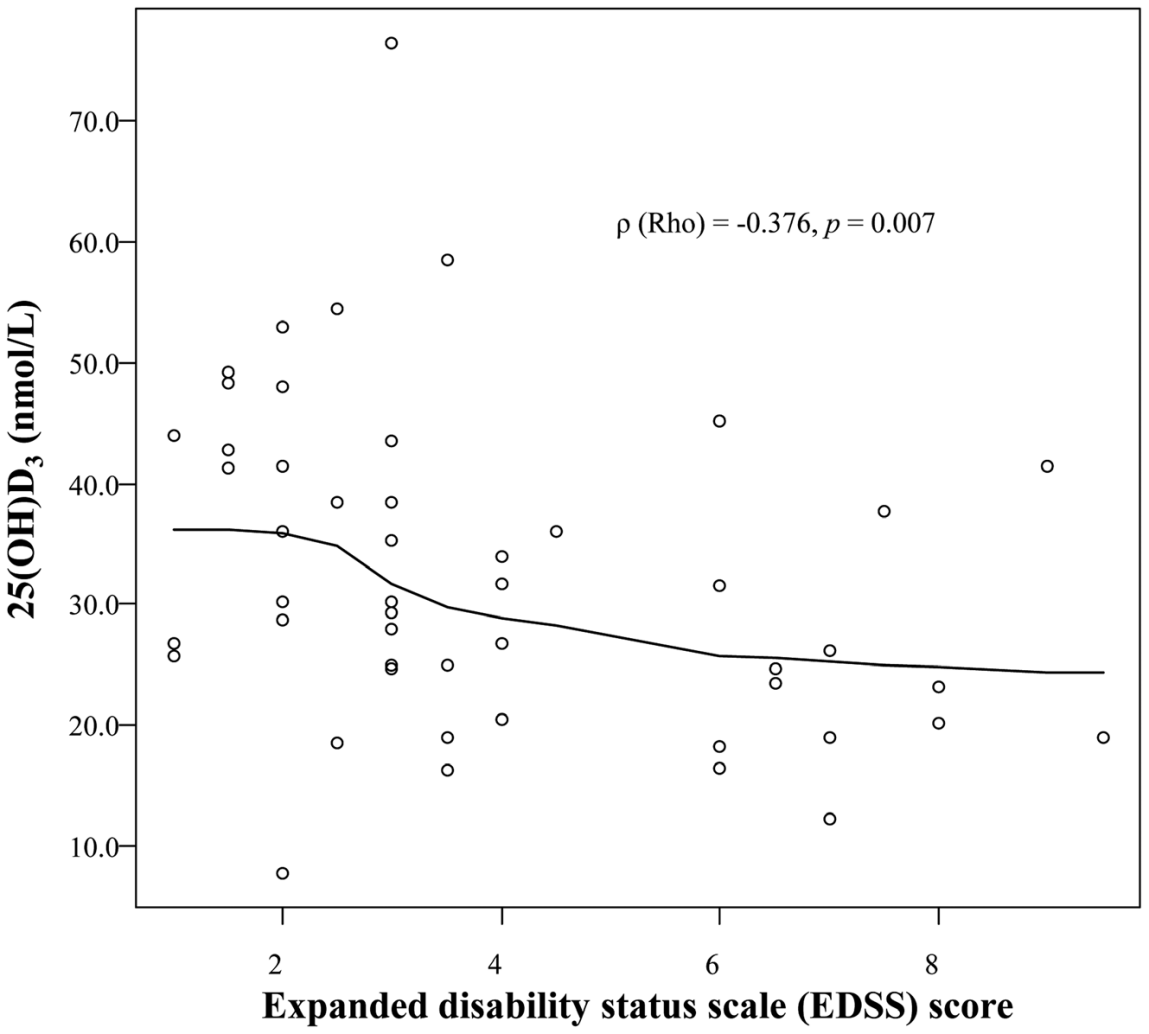
The relationship of 25-hydro 25-hydroxyvitamin D_3_ (25(OH)D_3_) levels and expanded disability status scale (EDSS) score in patients with neuromyelitis optica spectrum disorder. *ρ* (Rho), Spearman correlation coefficient.

**Figure 3:**
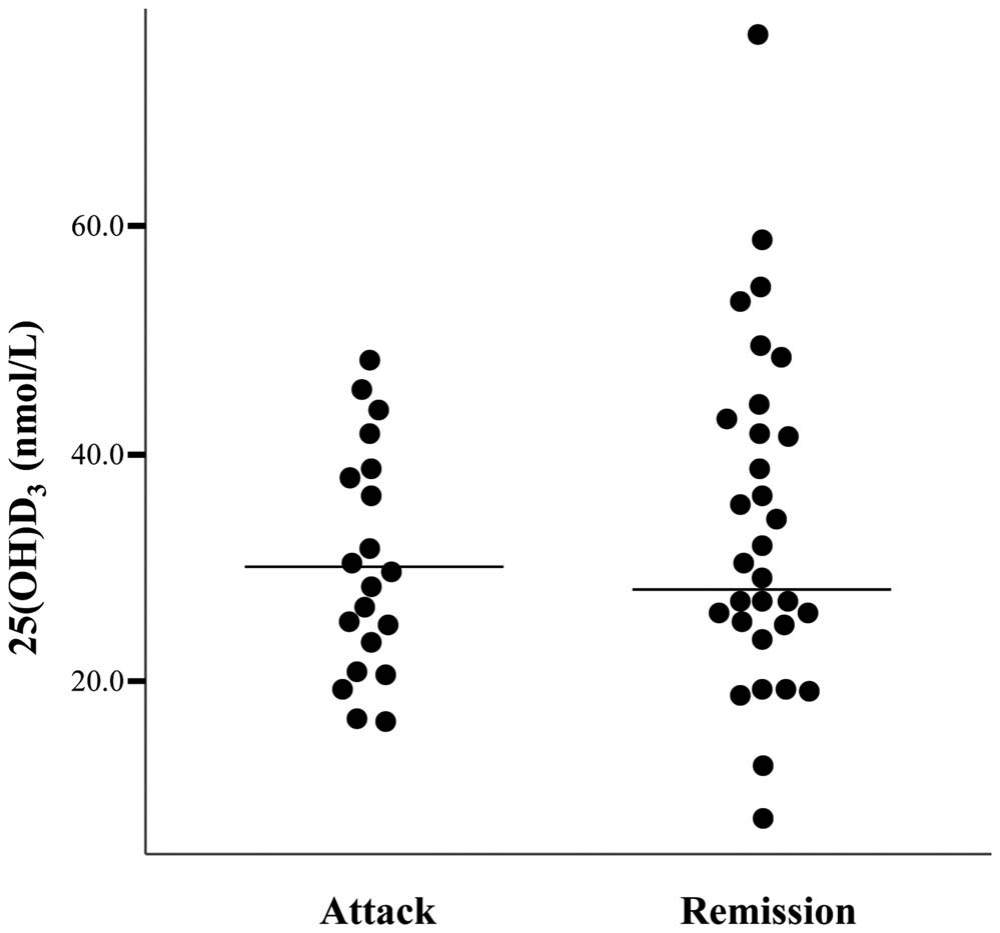
25-hydroxyvitamin D_3_ (25(OH)D_3_) levels in patients with neuromyelitis optica spectrum disorder during attack and in remission.

25(OH)D_3_ levels were inversely correlated with EDSS score (*ρ* = −0.37639, *p* = 0.007) and this persisted after adjustment for other factors, such as age, sex, BMI, season, prednisolone or azathioprine use, and disease duration (*ρ* = −0.38267, *p* = 0.012; Table 3 and Figure 2). All patients providing blood samples during an attack showed vitamin D deficiency (25(OH)D_3_ levels <50 nmol/L), compared with 27 (87.1%) patients of 31 taken during remission (*Fisher's exact test*, *p* = 0.145; Table S1). However, 25(OH)D_3_ levels were not associated with ARR (*ρ* = −0.13956, *p* = 0.372; Table S2). Multivariable logistic regression analysis also showed that 25(OH)D_3_ levels were not different between patients experiencing an attack and those who were in remission (*β* = −0.3653, *p* = 0.254) (Figure 3). Moreover, the location of lesion (optic nerve, spinal cord, or brain) did not correlate with 25(OH)D_3_ levels in our patients (*p* = 0.811; Table S3).

**Table 3:** Spearman's correlation analysis for the relationship between 25-hydroxyvitamin D_3_ (25(OH)D_3_) levels and expanded disability status scale (EDSS) score in patients with NMOSD.

Correlation values		25(OH)D_3_ & EDSS
Spearman	Rho (*ρ*)	−0.37639
	*p*-value	0.0065
Partial Spearman	Rho (*ρ*)	−0.38267
	*p*-value	0.0124

Abbreviations: 25(OH)D_3_, 25-hydroxyvitamin D_3_; EDSS, expanded disability status scale; Rho(*ρ*), Spearman correlation coefficients.

## Discussion

This is the first study to analyze vitamin D levels in patients with NMOSD and assess the relationship between vitamin D and clinical parameters. We found that patients with NMOSD had significantly lower levels of 25(OH)D_3_ than age, and sex matched control subjects as previously reported in other autoimmune disorders such as MS or SLE [19], [20]. In healthy controls, we found a high prevalence of vitamin D deficiency (66.2%), which is consistent with a previous study of healthy Korean subjects, which reported vitamin D deficiency (<20 ng/ml) in 47.3% of males and 64.5% of females [21]. Our result indicates that patients with NMOSD should be considered at high risk for vitamin D deficiency and, if required, supplementation might be beneficial in these patients. The relationship between NMO and vitamin D deficiency is unclear. It could reflect the influence of vitamin D deficiency on NMOSD risk or, conversely, the limited sunlight exposure as a result of immobilization. Since our study was not prospective, we could not analyze lifestyles such as outdoor activity, sun exposure, occupation, or diet, which could influence serum levels of vitamin D.

Recently, it was reported that vitamin D levels were lower in patients with recurrent transverse myelitis (TM) (n = 33; 61% AQP4-Ab positive) than in those with monophasic TM (n = 44; 0% AQP4-Ab positive) [13]. This paper showed that vitamin D levels were not correlated with length of time between disease onset and blood draw in patients with inflammatory spinal cord disease, of whom some were positive for NMO-IgG [13]. However, 25(OH)D_3_ levels were related to disease duration in our patients, and in MS patients [22]. In MS, serum levels of 25(OH)D_3_ were positively correlated with the capacity of Tregs to suppress T cell proliferation [23], which was also correlated with disease duration [24]. However, the relationship of vitamin D and Treg remains to be elucidated in NMO. Additionally, the possible role of vitamin D in MS is now widely spoken of and the MS patients could have taken vitamin D supplements during the disease course [25], which may explain higher vitamin D levels in MS patients with longer disease duration. Any data about a potential protective effect of vitamin D is lacking in NMO.

Interestingly, vitamin D levels were inversely correlated with EDSS scores in our patients. Previous studies of patients with MS have shown that vitamin D was associated with disease disability (EDSS), relapse risk, or magnetic resonance imaging (MRI) outcome [8], [22], [26]. In addition, in patients with undifferentiated connective tissue disorders, vitamin D deficiency was associated with the progression into well-defined connective tissue disorders such as RA or SLE [27]. It is possible that NMOSD patients with higher levels of disability showed lower 25(OH)D_3_ levels because lifestyle changes led to less outdoor activity and inadequate sun exposure after the disease onset. In addition, daily oral glucocorticoid use during the disease course may be associated with lower 25(OH) vitamin D levels, as observed in SLE patients [28]. However, our results demonstrated that vitamin D levels were higher in patients with longer disease duration. Therefore, we suggest that serum vitamin D levels are reflective of disease disability and/or vitamin D deficiency may have a detrimental effect on disease course in NMOSD. By contrast, we did not find evidence that serum vitamin D plays a role in NMOSD disease activity, which is inconsistent with findings observed in other autoimmune disorders. In MS, low serum vitamin D_3_ levels were associated with attack and also predicted new brain MRI findings [22], [29] and the association between vitamin D and disease activity was also found in SLE and RA [30], [31]. Recent studies indicated that interleukin-6 (IL-6) is involved in the pathogenesis of NMO [32], [33], and blockade of the IL-6 pathway, by tocilizumab, a humanized monoclonal anti-IL-6 receptor antibody, has been suggested as a new therapy for NMOSD [34]. It is used in patients with RA [35], where others have shown that 1,25(OH)_2_D_3_ had a similar effect, by reducing the secretion of IL-6 in peripheral blood mononuclear cells of patients [36]. Thus, future studies on IL-6 may elucidate further our understanding of vitamin D levels and NMOSD disease activity.

NMO is distinct from MS in clinical, epidemiological, radiological, cerebrospinal fluid and serological features [16]. In particular, the prevalence of MS is higher in high-latitude regions, where sunshine, required for the synthesis of vitamin D, is lacking, which suggests that vitamin D deficiency could be a risk for MS. By contrast, geographic variations were not observed in the incidence or prevalence of NMO between regions [37]. In addition, pathologic studies do not indicate that antibodies to specific membrane targets like AQP4 are primarily involved in the immunopathologic process of MS, as opposed to NMO [38], [39]. The roles of B and T cells in the pathogenic cascades of MS and NMO are considered to be fundamentally different [40] and they are immunologically distinguishable diseases, with different treatments required [41]. Therefore, the potential role of vitamin D in NMO and NMOSD should be different from that of MS, which needs to be investigated in further studies.

This study has several limitations. We only assessed patients treated at a single hospital, even though NMO and NMOSD are rare; the number of subjects was small [41]. In addition, we examined single ethnic population, which can lead to unintentional bias. Genetic factors such as vitamin D receptor gene polymorphism may be associated with vitamin D levels, as in MS and SLE [42], [43]. Finally, individual lifestyles affecting vitamin D levels were not analyzed in this study; therefore, the possibility of reverse causality between vitamin D levels and disease disability cannot be completely ruled out.

In conclusion, patients with NMOSD can be of high risk for vitamin D deficiency and we recommend the screening of vitamin D levels in these patients. The association of vitamin D levels and disease disability implies that vitamin D may have a modulating effect on disease course in NMOSD, although the causal-effect relationship is not certain. Currently, there are several randomized, placebo-controlled, double-blind trials investigating the clinical effect of vitamin D in MS patients, although so far available results are inconclusive [44]. Further prospective interventional studies are needed to elucidate the role of vitamin D in patients with NMOSD.

## Supporting Information

Table S1 **The frequency of vitamin D deficiency between patients during an attack (N = 20) and remission (N = 31), analyzed by Fisher's exact test.**
(DOCX)

Table S2 **Spearman's correlation analysis for the relationship between 25-hydroxyvitamin D_3_ (25(OH)D_3_) levels and annualized relapse rate (ARR) in patients with NMOSD.**
(DOCX)

Table S3 **Generalized estimating equation model for the relationship between 25-hydroxyvitamin D_3_ (25(OH)D_3_) levels and the location of lesion.**
(DOCX)
