# Supplementary material for: Low Levels of Vitamin D in Neuromyelitis Optica Spectrum Disorder: Association with Disease Disability
Source: PLoS One. 2014 Sep 11;9(9):e107274. doi: 10.1371/journal.pone.0107274 (PMC4161425; doi:10.1371/journal.pone.0107274)
Supplement: Table S1 — The frequency of vitamin D deficiency between patients during an attack (N = 20) and remission (N = 31), analyzed by Fisher's exact test. (DOCX) [file pone.0107274.s001.docx]

**Table S1: The frequency of vitamin D deficiency between patients during an attack (N=20) and remission (N=31), analyzed by Fisher’s exact test.**

|  |  | |  |  |  |
| --- | --- | --- | --- | --- | --- |
|  | No.(%) of vitamin D deficiency (<50 nmol/L) | | |  |  |
|  | Yes | No | | Total | p-value |
| NMOSD patients during an attack | 20 (100) | 0 (0) | | 20 | 0.145 |
| NMOSD patients during remission | 27 (87.1) | 4 (12.9) | | 31 |  |

No, number; NMOSD, neuromyelitis optica spectrum disorder
