# Supplementary material for: Low Levels of Vitamin D in Neuromyelitis Optica Spectrum Disorder: Association with Disease Disability
Source: PLoS One. 2014 Sep 11;9(9):e107274. doi: 10.1371/journal.pone.0107274 (PMC4161425; doi:10.1371/journal.pone.0107274)
Supplement: Table S2 — Spearman's correlation analysis for the relationship between 25-hydroxyvitamin D3 (25(OH)D3) levels and annualized relapse rate (ARR) in patients with NMOSD. (DOCX) [file pone.0107274.s002.docx]

**Table S2.** Spearman’s correlation analysis for the relationship between 25-hydroxyvitamin D_3_ (25(OH)D_3_) levels and annualized relapse rate (ARR) in patients with NMOSD.

| Correlation values | | 25(OH)D_3_ & ARR |
| --- | --- | --- |
| Spearman | Rho (*ρ*) | -0.1832 |
|  | *p*-value | 0.1982 |
| Partial Spearman | Rho (*ρ*) | -0.13956 |
|  | *p*-value | 0.3721 |

Abbreviations: 25(OH)D_3_, 25-hydroxyvitamin D_3_; ARR, annualized relapse rate; Rho(*ρ*), Spearman correlation coefficients
