# Supplementary material for: Low Levels of Vitamin D in Neuromyelitis Optica Spectrum Disorder: Association with Disease Disability
Source: PLoS One. 2014 Sep 11;9(9):e107274. doi: 10.1371/journal.pone.0107274 (PMC4161425; doi:10.1371/journal.pone.0107274)
Supplement: Table S3 — Generalized estimating equation model for the relationship between 25-hydroxyvitamin D3 (25(OH)D3) levels and the location of lesion. (DOCX) [file pone.0107274.s003.docx]

**Table S3. Generalized estimating equation model for the relationship between 25-hydroxyvitamin D_3_ (25(OH)D_3_) levels and the location of lesion.**

| Variable | Estimate | Standard Error | 95% Confidence Limits for estimate | | Z | p- value |
| --- | --- | --- | --- | --- | --- | --- |
| Group (Location of lesion) | |  |  |  |  |  |
| Optic nerve | -1.1399 | 2.5836 | -6.9307 | 4.651 | -0.44 | 1.000* |
| Spinal cord | -1.3192 | 2.0002 | -5.8023 | 3.164 | -0.66 | 1.000* |
| Brain | Reference | - | - | - | - | - |
| Disease duration^†^(years) | -0.0003 | 0.0003 | -0.0009 | 0.0002 | -1.24 | 0.2135 |
| Season |  |  |  |  |  |  |
| Spring | -3.3068 | 1.635 | -7.2196 | 0.6061 | -2.02 | 0.1293* |
| Summer | 1.0359 | 2.1831 | -4.1888 | 6.2606 | 0.47 | 1.000* |
| Fall | 0.1215 | 3.6143 | -8.5285 | 8.7715 | 0.03 | 1.000* |
| Winter | Reference | - | - | - | - | - |
| Oral prednisolone use |  |  |  |  |  |  |
| Yes | 1.4851 | 2.4962 | -3.4075 | 6.3776 | 0.59 | 0.5519 |
| No | Reference | - | - | - | - | - |
| Azathioprine use |  |  |  |  |  |  |
| Yes | -1.6322 | 1.5328 | -4.6363 | 1.372 | -1.06 | 0.2869 |
| No | Reference | - | - | - | - | - |
| Age (years) | -0.029 | 0.0767 | -0.1792 | 0.1213 | -0.38 | 0.7054 |
| Body mass index | 0.2 | 0.1742 | -0.1414 | 0.5414 | 1.15 | 0.2509 |
| Sex |  |  |  |  |  |  |
| Male | 1.9384 | 2.439 | -2.842 | 6.7187 | 0.79 | 0.4268 |
| Female | Reference | - | - | - | - | - |

* P-values were corrected by Bonferroni's method due to multiple testing.

^†^ Disease duration, interval from onset to blood sampling
